# Xanthohumol, a Prenylated Flavonoid from Hops, Induces Caspase-Dependent Degradation of Oncoprotein BCR-ABL in K562 Cells

**DOI:** 10.3390/antiox8090402

**Published:** 2019-09-16

**Authors:** Xuxiu Lu, Jiajia Geng, Jinman Zhang, Jinlai Miao, Ming Liu

**Affiliations:** 1 Key Laboratory of Marine Drugs, Ministry of Education, School of Medicine and Pharmacy, Ocean University of China, Qingdao 266003, China; xuxiulu@126.com (X.L.); 15232156167@163.com (J.G.); hdszhjm@126.com (J.Z.); 2 Laboratory for Marine Drugs and Bioproducts of Qingdao National Laboratory for Marine Science and Technology, Qingdao 266237, China; 3 Key Laboratory of Marine Bioactive Substances, First Institute of Oceanography, MNR, Qingdao 266061, China; 4 State Key Laboratory for Chemistry and Molecular Engineering of Medicinal Resources, Guangxi Normal University, Guilin 541001, China

**Keywords:** K562, xanthohumol, BCR-ABL, degradation, autophagy, caspase

## Abstract

BCR-ABL oncoprotein drives the initiation, promotion, and progression of chronic myelogenous leukemia (CML). Tyrosine kinase inhibitors are the first choice for CML therapy, however, BCR-ABL mediated drug resistance limits its clinical application and prognosis. A novel promising therapeutic strategy for CML therapy is to degrade BCR-ABL using small molecules. Antioxidant xanthohumol (XN) is a hop-derived prenylated flavonoid with multiple bioactivities. In this study, we showed XN could inhibit the proliferation, induce S phase cell cycle arrest, and stimulate apoptosis in K562 cells. XN degraded BCR-ABL in a concentration- and time-dependent manner, and the involved degradation pathway was caspase activation, while not autophagy induction or ubiquitin proteasome system (UPS) activation. Moreover, we revealed for the first time that XN could inhibit the UPS and autophagy in K562 cells, and the inhibitory effect of XN on autophagy could attenuate imatinib-induced autophagy and enhance the therapeutic efficiency of imatinib in K562 cells. Our present findings identified XN act as a degrader of BCR-ABL in K562 cells, and XN had potential to be developed as an alternate agent for CML therapy.

## 1. Introduction

Chronic myelogenous leukemia (CML) is characterized by the BCR-ABL fusion protein with constitutive kinase activity [1,2]. This aberrant tyrosine kinase is mainly responsible for malignant transformation by activating multiple signal transduction pathways [3,4]. The tyrosine kinase inhibitor imatinib has been developed with remarkable effects in CML treatment; however, point mutations in the tyrosine kinase domain of BCR-ABL and/or high expression of BCR-ABL mRNA usually induce resistance to tyrosine kinase inhibitors [5,6,7,8]. Therefore, novel methods to overcome this resistance are required. Increasing evidences suggest that inducing oncoprotein degradation is a novel and effective therapeutic approach [9,10], and small compounds are promising candidates for reducing BCR-ABL expression and have been regarded as potential drug candidates for CML treatment. For example, oridonin and non-anasamycin compound EC141 induce BCR-ABL degradation via ubiquitin proteasome system (UPS) [11,12] and inhibit the growth of Ph^+^ leukemia cells. Arsenic trioxide (As_2_O_3_) autophagically degrades BCR-ABL and exhibits potent antileukemia activity [13]. Gambogic acid decreases BCR-ABL by activating the caspase system both in imatinib resistant and sensitive CML cells [14], while platinum pyrithione downregulates BCR-ABL through inhibiting BCR-ABL transcription, as well as degrading BCR-ABL caspase dependently [15]. All these studies strongly encourage a new therapeutic strategy for targeting BCR-ABL expression to improve the CML therapeutic outcomes.

**Figure 1:**
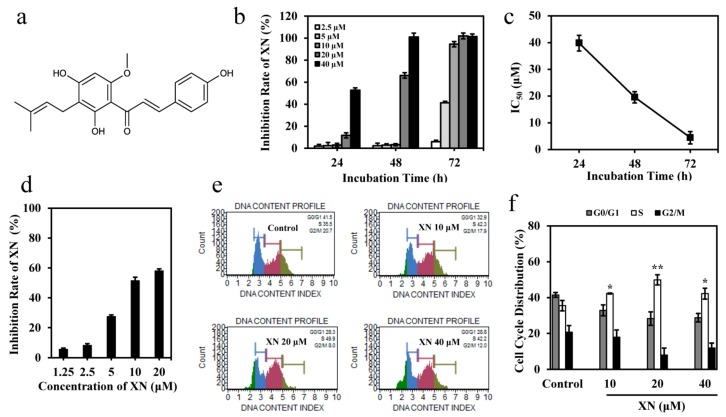
Xanthohumol (XN) decreases cell viability of K562 cells. (**a**) Chemical structure of XN. (**b**) Proliferation inhibition rates (%) of XN against K562 cells. K562 cells were treated with XN (0–40 μM) for 24–72 h. Cell viability was subjected to MTT assay. (**c**) The IC_50_ values of XN against K562 cells at 24–72 h, respectively. (**d**) Proliferation inhibition rates (%) of XN against K562/ADR cells. K562/ADR cells were treated with XN at concentrations of 0–20 μM for 72 h, and then subjected to MTT assay. (**e**) XN induces cell cycle arrest at S phase. K562 cells were treated with XN (0–40 μM) for indicated time and the cell cycle distribution was measured using Muse Cells Analyzer. (**f**) The bar graph depicts the percentage of each cell cycle phase of K562 cells in the absence or presence of XN. Values are expressed as mean ± SD of three independent experiments. * *p* < 0.05, ** *p* < 0.01, versus control.

Antioxidant xanthohumol (XN, Figure 1a) is the most abundant prenylated flavonoid in hops used in beer production, and beer is the principle dietary source of XN [16,17]. XN has a good safety profile [18], and exhibits numerous beneficial effects including anticancer, hypoglycemic, anti-inflammatory, anti-obesity activities, and so on [19]. The anticancer effect interests extensive studies, and this activity can be associated with its antioxidant activity [20]. When XN isomerizes to the less antioxidant active isoxanthohumol (IXN), or modifies to other derivatives, XN showed the distinctive reduction in its antiproliferative activity [21,22]. Notably, the antileukemia effect of XN has been reported in several studies. For example, XN induced apoptosis of chronic myeloid leukemia cells by decrease of NF-κB activation [23]. XN also induced apoptosis and modulated BCR-ABL expression in K562 cells [8]. However, the antileukemia effect of XN and the underlying mechanism in the aspect of BCR-ABL expression has to be further investigated.

In the present study, we aimed to elucidate the anticancer activity of XN against human chronic myelogenous leukemia K562 cells in vitro, and to investigate the underlying mechanism. The effect of XN on the cell proliferation, cell cycle distribution, apoptosis, and the degradation of BCR-ABL in K562 cells were fully evaluated.

## 2. Materials and Methods

### 2.1. Reagents and Drug

XN (purity > 98%) was provided by Nanjing Spring and Autumn Biological Engineering Co., Ltd., Nanjing, China. Antibodies against C-ABL, phosphorylated C-ABL at Y245, cleaved caspase-3 (C-Cas3), cleaved caspase-9 (C-Cas9), cleaved PARP (C-PARP), LC3B, p62, Hsp70, and ubiquitin were purchased from Cell Signaling Technology (Boston, MA, USA). Z-VAD-fmk was obtained from Selleck Chemicals (Houston, TX, USA). MG132 and chloroquine (CQ) were obtained from Sigma-Aldrich (St. Louis, MO, USA). Muse™ Cell Cycle Kit and Muse^®^ Annexin V & Dead Cell Kit were purchased from Millipore (Billerica, MA, USA). Other reagents were purchased from Beyotime Biotechnology, Shanghai, China.

### 2.2. Cell Lines and Cell Culture

Human chronic myelogenous leukemia cell K562 and its adriamycin-resistant cell line K562/ADR were purchased from Shanghai Cell Bank, Chinese Academy of Science. Cells were cultured in Iscove’s Modified Dulbecco’s Medium (GIBCO, Grand Island, NY, USA) containing 10% fetal bovine serum (FBS) and 1% penicillin/streptomycin at 37 °C in a humidified incubator containing 5% CO_2_.

### 2.3. Cell Viability Assessment

Cell viability was determined by 3-(4,5-dimethylthiazol-2-yl)-2,5-diphenyltetrazolium bromide (MTT) method. Briefly, cells were seeded into 96-well plates (5000 cells each well) and treated with different concentrations of XN for the indicated time. Then MTT reagent was added to each well and incubated for 4 h. Acidic isopropanol (100 μL) was added into the reaction mixture and plates were further incubated overnight to dissolve the formazan product. Finally, the absorbance was measured at 570 nm using a microplate reader (BioTek, VT, USA).

### 2.4. Cell Cycle Analysis

K562 cells were seeded in six-well plates (5 × 10^5^ cells each well), and treated with progressive concentrations of XN for 24 h. The control group was treated with vehicle DMSO. Then cells were collected, washed, and fixed in 70% cold ethanol overnight at −20 °C. Cells were collected, washed, and stained with Muse™ cell cycle reagent (200 μL) for 30 min in the dark. The cell cycle distribution was detected with Muse Cell Analyzer (Millipore, Billerica, MA, USA).

### 2.5. Drug Combination and Calculation of Synergism

Cells were treated with XN, imatinib, alone, or both of them for indicated concentrations. MTT assays were performed after incubation for 72 h. The concentration-response data were analyzed by the medium-effect method, and the synergistic effect of multiple drugs was calculated by the definition of Chou and Talalay [24]. The combination index (CI) reflecting the synergism of two drugs was calculated by Calcusyn (Biosoft, Cambridge, UK). The CI values of <1, 1, and >1 indicate synergistic, additive, and antagonistic effects, respectively.

### 2.6. Westerrn Blotting Assay

Cells were seeded in six-well plates (5 × 10^5^ cells each well), and incubated with different reagents or treated with different time. Then cells were collected, washed, and lysed with loading buffer (0.125 M Tris-HCl, 5% 2-mercaptoethanol, 30 mg/mL sodium dodecyl sulfate (SDS), 10% glycerol, 0.5 mg/mL bromophenol blue) for 45 min at 4 °C. The lysates were boiled 15 min and stored at −20 °C. Protein samples were separated by electrophoresis on 6–12% SDS-PAGE and transferred to membranes. The membrane was blocked in 5% skim milk for 1 h and incubated with indicated primary antibodies overnight at 4 °C. Then the membranes were incubated with HRP-secondary antibody at room temperature and detected by FluorChem E (Protein Simple, San Jose, CA, USA).

### 2.7. Cychloheximide (CHX) Chase Assay

BCR-ABL protein stability was detected by CHX chase assay. Briefly, K562 cells were treated with CHX (20 μg/mL) in the absence or presence of XN (20 or 40 μM) for the indicated time. Cells were collected, washed, and BCR-ABL levels were detected by Western blotting, and the density ratio to control was analyzed.

### 2.8. Cell Apoptosis Analysis

Apoptosis was determined by Annexin V-PE/7-AAD staining. Briefly, K562 cells were treated with XN, imatinib, alone, or in combination for 24 h. Then cells were centrifuged at 1000 rpm at 4 °C for 5 min, washed with ice-cold phosphate-buffered saline (PBS). Following this, cells were suspended in Iscove’s Modified Dulbecco’s Medium containing 1% FBS and stained with Muse^®^ Annexin V & Dead Cell Kit (Millipore, Billerica, MA, USA) for 20 min at room temperature in the dark, and finally detected with Muse Cell Analyzer.

### 2.9. Statistical Analysis

The results shown in this study were represented as the mean ± SD. Comparisons between the groups were assessed by Student’s *t*-test and *p* < 0.05 were defined statistically significant.

## 3. Results

### 3.1. XN Inhibits the Viability of K562 Cells

The cytotoxicity of XN on K562 cells were determined by MTT assay. The results indicated XN inhibited the proliferation of K562 cells in a time- and concentration-dependent manner (Figure 1b), with IC_50_ values of 39.82, 19.56, and 4.43 μM at 24, 48, and 72 h, respectively (Figure 1c). In addition, we found XN had moderate effect on drug resistant K562/ADR cells, and the inhibition effect of XN at 20 μM was about 58% after 72 h of treatment (Figure 1d). To further study the mechanisms leading to cell death and growth inhibition following XN treatment, we analyzed the effect of XN on the cell cycle distribution of K562 cells, and the result showed XN significantly induced cell cycle arrest at S phase, with decrease in G0/G1 and G2/M phases, after treatment for 24 h (Figure 1e–f). These data suggested that XN inhibited proliferation and induced cell cycle arrest in K562 cells.

### 3.2. XN Inhibits Oncoprotein BCR-ABL Expression

**Figure 2:**
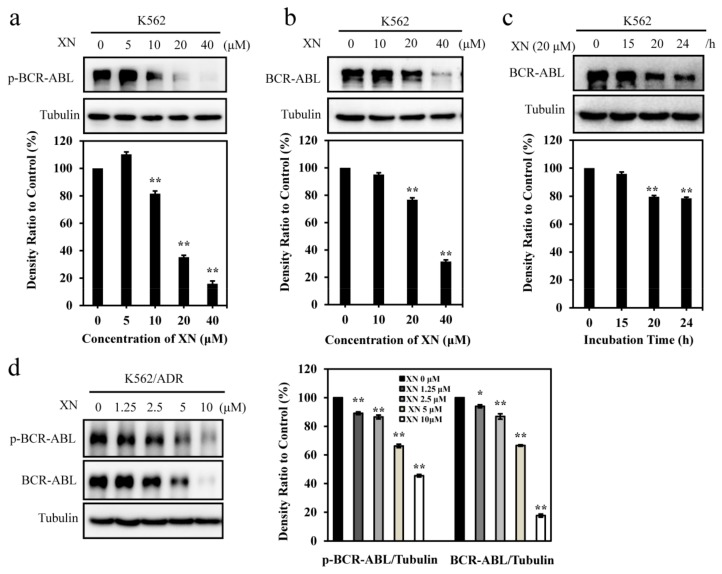
XN decreases the phosphorylated and the total protein level of BCR-ABL. (**a**) XN inhibits the phosphorylation of BCR-ABL. K562 cells were treated with XN (5, 10, 20, and 40 μM) for 24 h, and cell lysates were then immunoblotted to assess the changes of p-BCR-ABL and BCR-ABL. (**b**) XN concentration dependently decreases the protein level of BCR-ABL. K562 cells were treated with XN (0–40 μM) for 24 h. The BCR-ABL protein level was blotted by anti-ABL antibody. (**c**) XN time dependently decreases the protein level of BCR-ABL. K562 cells treated with 20 μM of XN at different time periods (15, 20, and 24 h). BCR-ABL expression was detected by Western blotting. (**d**) XN inhibits the phosphorylated and the total protein level of BCR-ABL in K562/ADR cells. K562/ADR cells were treated with XN (1.25–10 μM) for 24 h. The p-BCR-ABL and BCR-ABL were determined by Western blotting. Histograms show the relative abundance of p-BCR-ABL or BCR-ABL to the control group. Data are presented as mean ± SD for three independent experiments. * *p* < 0.05, ** *p* < 0.01, versus control.

Oncoprotein BCR-ABL contributes to CML initiation and maintenance [25], thus, we next investigated the effect of XN on BCR-ABL. First, we detected the phosphorylation of BCR-ABL and the results showed that a relatively higher concentration (>10 μM) of XN could inhibit the phosphorylation of BCR-ABL (Figure 2a). More interestingly, we found XN significantly inhibited the protein level of BCR-ABL in a concentration- (Figure 2b) and time- (Figure 2c) dependent manner in K562 cells. Similarly, XN could also inhibit the phosphorylation of BCR-ABL and decrease the total level of BCR-ABL in K562/ADR cells (Figure 2d). These results suggested that XN could affect both the phosphorylation and the total levels of BCR-ABL.

### 3.3. XN Promotes the Degradation of BCR-ABL Oncoprotein

**Figure 3:**
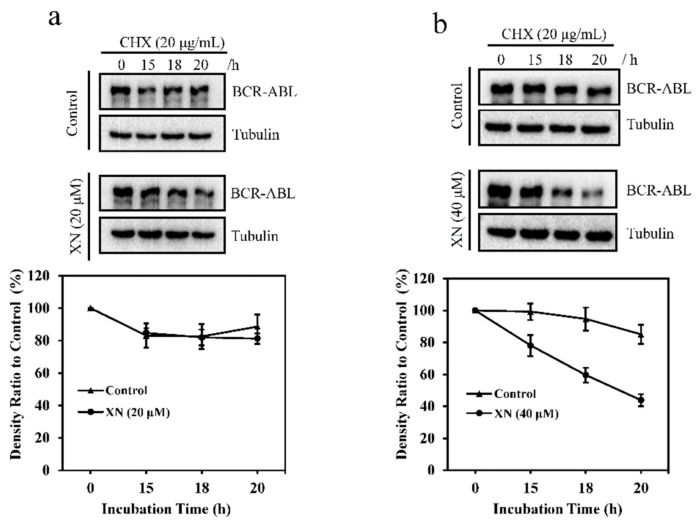
XN affects BCR-ABL protein stability. K562 cells were incubated with cychloheximide (CHX, 20 μg/mL) in the absence or presence of 20 (**a**) and 40 μM XN (**b**) for 0–20 h. Then the cells were harvested and BCR-ABL levels were examined by Western blotting, and the relative density of BCR-ABL was analyzed and shown as histograms. Data are presented as mean ± SD for three independent experiments.

To determine the mechanisms underlying XN-induced down-regulation of BCR-ABL, we investigated the effect of XN on BCR-ABL protein stability by using the protein biosynthesis inhibitor CHX. As shown in Figure 3a (upper panel), in the presence of CHX, BCR-ABL level decreased with the incubation time of CHX, while co-treatment with XN (20 μM) and CHX, BCR-ABL showed a faster degradation (Figure 3a, middle panel) as compared to cells treated with CHX alone. Higher concentration of XN (40 μM) accelerated the degradation of BCR-ABL more obviously (Figure 3b), suggesting that XN induced BCR-ABL degradation.

### 3.4. XN Inhibits Autophagosome Maturation and Autophagy is not Involved in BCR-ABL Degradation

**Figure 4:**
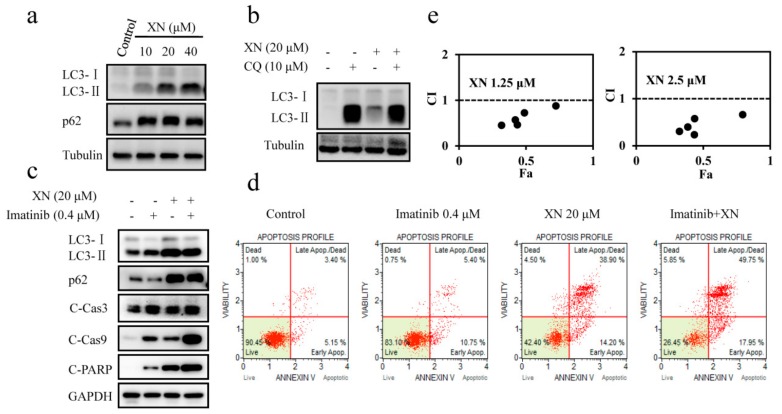
XN inhibits autophagosome maturation. (**a**) XN treatment alters autophagy-related proteins. K562 cells were treated with XN (0-40 μM) for 24 h. Cell lysates were immunoblotted with indicated antibodies. Tubulin was immunoblotted as a loading control. (**b**) XN inhibits autophagy at its late stage. K562 cells were treated with 10 μM chloroquine (CQ) and/or 20 μM XN for 24 h, and the expression of LC3 was determined by Western blotting. (**c**) XN alleviates imatinib mediated autophagy and increases imatinib-induced apoptosis. Cells were treated with 20 μM of XN in the presence or absence of imatinib (0.4 μM) for 24 h, the expression of LC3, p62, cleaved caspase-3 (C-Cas3), cleaved caspase9 (C-Cas9), and cleaved PARP (C-PARP) were determined by Western blotting. (**d**) Annexin V-PE/7-AAD staining in K562 cells treated with XN (20 μM) in the presence or absence of imatinib (0.4 μM) for 24 h were measured using Muse Cell Analyzer. (**e**) XN shows synergistic effect with imatinib. K562 cells were treated with 1.25 or 2.5 μM of XN with various concentrations of imatinib for 72 h. And values for the combination index (CI) was calculated using software package Calcusyn (Biosoft, Cambridge, UK), which interpreted as follows: >1 antagonism, <1 synergism, and =1 additive. Fa represented the fractions of the affected cells (killed). All experiments were performed in three replicates (n = 3).

Since autophagic degradation is one of the BCR-ABL degradation pathways [26], next we attempted to identify whether XN-induced degradation of BCR-ABL had a relationship with autophagy. Our result indicated that XN induced up-regulation of LC3-II expression in a concentration-dependent manner (Figure 4a). The increased LC3-II level may originate from increased formation of autophagosomes or impaired maturation of autophagosomes. To further distinguish these two possibilities, we examined the expression level of p62, which is degraded by autophagy pathway and accumulates when autophagy is impaired. As shown in Figure 4a, XN increased the expression of p62 significantly, indicating that the increased LC3-II level induced by XN was the consequence of attenuated maturation of autophagosomes, and thus indicating XN inhibited autophagy. To further confirm that XN blocked maturation of autophagosomes, we next analyzed LC3-II levels in XN-treated K562 cells in the presence of autophagy inhibitor chloroquine (CQ), which prevents autophagosome-lysosome fusion and blocks autophagic degradation [27]. If XN blocks autophagy, similar levels of LC3-II in CQ- and CQ/XN-treated cells will be observed, while if XN induces autophagy, LC3-II expression will be higher in CQ/XN-co-treated cells than CQ treated alone. We found that both CQ and XN could increase the levels of LC3-II compared to the control group, however, there was no significant increase in CQ/XN-co-treated cells compared to CQ treated alone (Figure 4b), further confirming XN blocked autophagy and XN-induced LC3-II accumulation was not via autophagy induction, but autophagy inhibition. All these results suggested that XN inhibited autophagy and therefore the degradation of BCR-ABL was not via autophagic degradation.

Imatinib treatment induces protective autophagy in CML cells, which provides a survival mechanism to BCR-ABL-expressing cells and contributes to drugs resistance [28]. The effect of XN on inhibition of autophagy suggested it may inhibit imatinib-induced autophagy and improve the therapeutic efficacy of imatinib in treatment of CML. Thus, we first analyzed the role of XN in imatinib-induced autophagy. Imatinib treatment increased the ratio of LC3-II to LC3-I protein level and decreased the expression of p62, confirming imatinib induced autophagy (Figure 4c). However, in the presence of XN, imatinib-induced the decrease of the p62 recovered, indicating XN attenuated imatinib-mediated autophagy. As a consequence, the autophagy inhibition by XN enhanced the cell sensitivity to imatinib, evidenced by the increased expression of apoptosis markers C-Cas3, C-Cas9, and C-PARP (Figure 4c), as well as the higher percentage of apoptotic cells detected by Annexin V/7-AAD staining (Figure 4d), and also by the strong synergistic effect (CI < 1) in cell proliferation inhibition when these two agents used in combination (Figure 4e). Similarly, the synergistic effect was also found in K562/ADR cells (Figure S1). More importantly, XN could also inhibited imatinib-mediated autophagy in K562/ADR cells, consequently, apoptosis was significantly enhanced due to autophagy inhibition (Figure S2). These data collectively suggested that XN inhibited autophagy and autophagy is not responsible for XN-induced BCR-ABL degradation.

### 3.5. XN-Induced BCR-ABL Degradation is Caspase-Dependent

**Figure 5:**
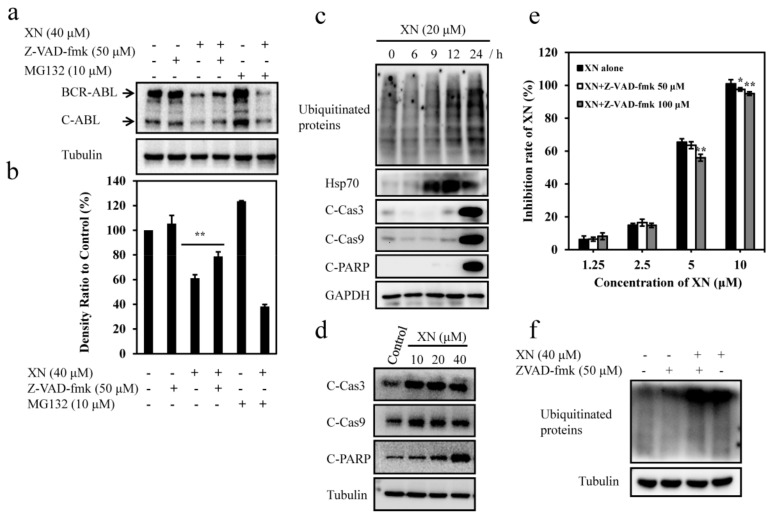
XN induces caspase-dependent BCR-ABL degradation and apoptosis in K562 cells. (**a**) XN decreases BCR-ABL protein in a caspase-dependent manner. K562 cells were untreated or pretreated for 1 h with 50 μM of pan caspase inhibitor Z-VAD-fmk or proteasome inhibitor MG132 (10 μM) either alone or in combination with XN (40 μM) for 24 h. Expressions of BCR-ABL were determined by Western blotting. Tubulin was used as a loading control. (**b**) Histograms show the relative intensity of BCR-ABL bands. Data are presented as mean ± SD (n = 3). ** *p* < 0.01, versus control. (**c**) XN inhibits the ubiquitin proteasome system activities in K562 cells. Cells are treated with 20 μM of XN for different time periods (6, 9, 12, and 24 h), followed by detection of ubiquitinated proteins, Hsp70, C-Cas3, C-Cas9, and C-PARP. (**d**) XN induces caspase activation and apoptosis. K562 cells were incubated with indicated concentrations of XN for 24 h, and cell lysates were then immunoblotted to assess the changes of C-Cas3, C-Cas9 and C-PARP. (**e**) Pan caspase inhibitor Z-VAD-fmk attenuates XN-induced cell death. K562 cells were pretreated with or without Z-VAD-fmk for 1 h followed by incubation with various concentrations of XN for 72 h, and then cells were subjected to MTT assay. Data are expressed as mean ± SD (n = 3). * *p* < 0.05, ** *p* < 0.01, versus XN alone. (**f**) Pan caspase inhibitor Z-VAD-fmk has no effect on ubiquitinated proteins. Cells were pretreated with or without Z-VAD-fmk for 1 h, followed by incubation XN (20 μM) for 24 h, and ubiquitinated proteins were detected by Western blotting.

Previous studies have reported that both ubiquitin proteasome system (UPS) and caspase activation were involved in the degradation of BCR-ABL [14,29]. We then investigated whether XN-induced BCR-ABL degradation had a relationship with UPS and caspase activation. To identify whether UPS was involved in the XN-mediated degradation of BCR-ABL, we pretreated K562 cells with proteasome inhibitor MG132 (10 μM) and further incubated with XN for 24 h, but found no attenuation on BCR-ABL degradation (Figure 5a,b), suggesting UPS activation was not responsible for BCR-ABL degradation. Unexpectedly, we found XN treatment induced marked increases of ubiquitinated proteins with proteasome substrate protein Hsp70 increased (Figure 5c), indicating XN inhibited proteasome activity. In addition, this is also consistent with our observation that UPS inhibitor MG132 did not reverse the decrease of BCR-ABL protein level (Figure 5a,b). We then observed that XN stimulated significant caspase 3 and 9 activation and therefore induced cleavage of PARP (Figure 5d), moreover, XN-induced BCR-ABL degradation was inhibited significantly after 24 h of treatment with pan caspase inhibitor Z-VAD-fmk (50 μM) (Figure 5a,b); simultaneously, XN-stimulated cell death was inhibited in the presence of Z-VAD-fmk (Figure 5e). These results demonstrated that XN-induced caspase activation was required for the degradation of BCR-ABL and cell death. However, Z-VAD-fmk did not attenuate ubiquitinated proteins accumulation (Figure 5f), moreover, apoptosis-specific C-Cas3, C-Cas9, and C-PARP was not obvious until 24 h of XN treatment, while the accumulation of ubiquitinated proteins and proteasome substrate Hsp70 were observed earlier than the appearance of apoptosis-specific C-Cas3, C-Cas9, and C-PARP (Figure 5c), suggesting XN-induced proteasome inhibition was in the upstream of caspase activation.

### 3.6. XN-Induced Caspase Activation and BCR-ABL Degradation were Enhanced by Autophagy Inhibitor CQ

**Figure 6:**
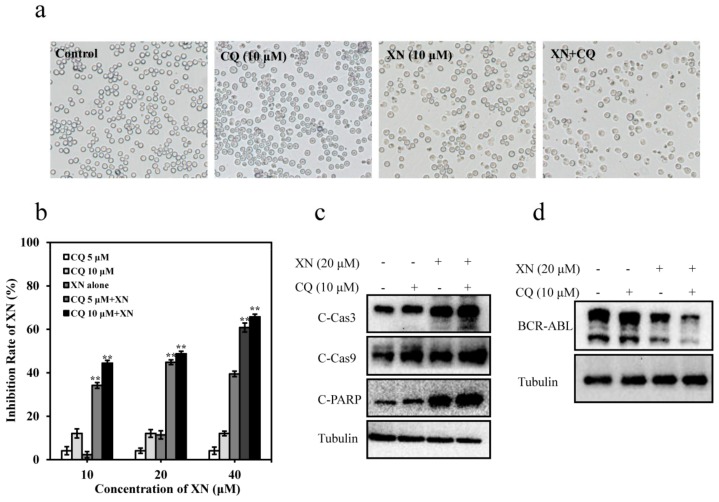
Autophagy inhibitor chloroquine (CQ) potentiates XN-induced caspase activation and BCR-ABL degradation. (**a**) Cell density and morphology of K562 treated with 10 μM of XN in the presence or absence of CQ (10 μM) for 24 h. (**b**) CQ enhances XN-induced cytotoxicity in K562 cells. Cells were treated with varying concentrations of XN for 24 h in the presence or absence of CQ (5 or 10 μM), and then subjected to MTT assay. Data are presented as mean ± SD of three independent experiments, and ** *p* < 0.01, versus XN alone. (**c**) Effect of CQ and XN on C-Cas3, C-Cas9 and C-PARP proteins. K562 cells treated with XN (20 μM) and CQ (10 μM) alone, or in combination, for 24 h. Cell lysates were analyzed with immunoblotting. Tubulin was used as loading control. (**d**) CQ accelerated XN-induced caspase-dependent degradation of BCR-ABL. K562 cells were pretreated with CQ for 1 h and then incubated with or without XN (20 μM) for further 24 h. BCR-ABL was examined by Western blotting.

In our present work, we found that CQ alone did not obviously affect K562 cell death; however, CQ could significantly enhance the activity of XN, showing more inhibition in the cell density (Figure 6a) and proliferation in K562 cells (Figure 6b). It has been proven that autophagy inhibitor CQ could induce caspase activation [30]. As expected, both CQ and XN induced activation of C-Cas3 and C-Cas9, and CQ/XN combination also resulted in a higher level of C-Cas3, C-Cas9, and C-PARP compared to either treatment alone, indicating CQ enhanced the XN-induced caspase activation, and further induced stronger apoptosis (Figure 6c). As we have concluded that the degradation of BCR-ABL required caspase activation, after enhancing the caspase activation by CQ, we also observed that XN-induced BCR-ABL degradation was accelerated significantly in the presence of CQ (Figure 6d), confirming caspase-dependent BCR-ABL degradation was enhanced by CQ. These data confirmed BCR-ABL degradation induced by XN was caspase-dependent and also revealed that autophagy inhibition would strengthen XN-induced caspase activation and degradation of BCR-ABL.

## 4. Discussion

CML is highly dependent on the existence of BCR-ABL [31], hence, downregulating BCR-ABL oncoprotein by small molecule compounds, via mRNA level or protein level, may be applied as a promising therapies for CML. The greatest advantage of this strategies may overcome the drug resistance resulted by the oncoprotein mutation and amplification [32]. Growing body of reports showed that natural products are a good source for the searching of oncoprotein degrading agents [11,14,33].

The health benefits of dietary antioxidant flavonoids have attracted increasing research interest nowadays and a number of evidences have indicated that flavonoids-rich food aids in the prevention and treatment of cancer. XN, the principal prenylated chalcone existing in the female inflorescences of hops and beer, has been proved to prevent and treat many kinds of cancers [17,22,34]. The underlying mechanisms of XN exerts its anticancer activity have been tried to identify, including inhibition of proliferation, attenuation of angiogenesis, inhibition of migration and induction of apoptosis in various cancer cells [20,35,36,37,38,39]. Both our present work and the previous report [8] indicated that XN showed antileukemia activities and could modulate BCR-ABL. S. Monteghirfo et. al. reported that XN showed antileukemia activities via modulating the expression level of BCR-ABL on mRNA level in K562 cells [8]. In addition to affect the BCR-ABL mRNA level, our current work for the first time demonstrated that relatively higher concentration of XN also affected both the activation of BCR-ABL kinase and the stability of BCR-ABL at protein level in K562 cells. In addition, the degradation of BCR-ABL was also observed in K562/ADR cells (Figure 2d). Therefore, the effective inhibition of BCR-ABL induced by XN could provide a targeted pathway for both sensitive CML as well as that already developing multidrug resistant. To the best of our knowledge, this was the first report to show that XN was effective in BCR-ABL degradation at protein level. The protein degradation activity of XN was similar to other prenylflavonoid compound (e.g., IXN), which induced sterol regulatory element-binding proteins transcription factors degradation via ubiquitin-proteasome-dependent pathway [40].

Next we investigated the mechanisms underlying XN-induced BCR-ABL degradation. Generally, autophagic degradation pathway [26], UPS pathway [29], and the caspase pathway [14,41] are the major pathways contributing to BCR-ABL degradation. First, we attempted to identify whether XN-induced degradation of BCR-ABL had a relationship with autophagy, however, we found XN inhibited autophagy in K562 cells, rather than inducing autophagy. Similarly, the autophagy inhibition was also observed in XN-treated A431 cell lines [42]. It has been reported that autophagy was inhibited by XN at the fusion of autophagosomes with lysosomes step in HL-60 cells [43]. Thus, autophagy was not the degradation way of XN-induced BCR-ABL degradation. Importantly, we found autophagy inhibition induced by XN could attenuate imatinib-induced protective autophagy and therefore enhanced imatinib-induced apoptosis (Figure 4, Figure S2). In this regard, XN could enhance the therapeutic efficacy of imatinib via alleviating imatinib-induced autophagy; on the other hand, XN itself could also eliminate oncoprotein BCR-ABL and induce apoptosis, suggesting XN, at least, could serve as an imatinib sensitizer and an oncoprotein degrader in the treatment of CML. Previous study have suggested flavonoids and its derivatives could be served as candidates of chemosensitizer, and this chemosensitizing function might be through inhibiting NF-κB, P-glycoproteins, ATR/ATM signaling, AKT, and increasing the expression of p53 protein [44,45]. For the first time, we found a novel mechanism by which XN enhanced K562 cells sensitivity to imatinib was related to autophagy inhibition and BCR-ABL degradation. Next, we investigated whether XN-stimulated BCR-ABL degradation had a relationship with UPS. We found that UPS was inhibited after XN treatment in K562 cells, evidenced by accumulated ubiquitinated proteins and increased expression level of proteasome substrate Hsp70, which was consistent with the previous report that XN inhibited the proteasome activity in HL-60 cells [43]. Furthermore, the proteasome inhibitor MG132 exerted no attenuation on BCR-ABL degradation (Figure 5a,b). Thus, we concluded that XN-stimulated BCR-ABL degradation was not due to UPS activation. In addition, XN-induced UPS inhibition occurred earlier than apoptosis-specific C-Cas3, C-Cas9, and C-PARP, moreover, pan-caspase inhibitor Z-VAD-fmk did not attenuate the accumulated ubiquitinated proteins, implying a possibility that XN-mediated UPS inhibition contributed to the activation of caspase pathway, which consequently degraded BCR-ABL. This was similar to bortezomib, which has been reported to inhibit the proteasome activity and subsequently induces caspase activation in BCR-ABL-expressing cells [46], however, further experiments are needed to prove our hypothesis. Furthermore, using Z-VAD-fmk, we revealed that XN induced caspase activation in K562 cells, which directly induced BCR-ABL degradation, as well as apoptosis in K562 cells (Figure 5a,b,e). XN treatment also induced remarkable caspase activation in other types of cancer, such as gastric cancer [36] and thyroid cancer [20]. This was similar with the XN derivatives dihydroxanthohumol (DXN) and tetrahydroxanthohumol (TXN), which possess improved anti-proliferative activity compared with XN and could also induce obvious caspase-mediated apoptosis [47]. Finally, using the autophagy inhibitor CQ, which could inhibit the autophagy and enhance the caspase activity [30], we found CQ enhanced XN-stimulated caspase activation and apoptosis (Figure 6c). Notably, more BCR-ABL degradation was observed in the presence of CQ (Figure 6d), further confirming caspase activation and autophagy have a close relationship with BCR-ABL degradation.

## 5. Conclusions

In summary, we confirmed that XN effectively inhibited K562 cells proliferation, arrested cell cycle, stimulated apoptosis, and decreased BCR-ABL oncoprotein level. For the first time, we revealed that XN could degrade BCR-ABL at protein level and the mechanisms underlying XN-stimulated BCR-ABL degradation was related to the caspase activation, rather than directly by UPS pathway or autophagy activation. Our work revealed an alternative strategy to downregulate BCR-ABL level via activating the caspase system. In addition, XN inhibited imatinib-induced autophagy and showed synergistic effect with imatinib in CML treatment, suggesting the potential combination administration with imatinib. Taken together, XN could degrade BCR-ABL and XN may have promising efficacy against human CML.

## Supplementary Materials

The following are available online at https://www.mdpi.com/2076-3921/8/9/402/s1, Figure S1: Evaluated the synergistic effect of XN combined with imatinib in K562/ADR cells. Figure S2: XN attenuates imatinib mediated autophagy and enhances apoptosis in K562/ADR cells.
