# Supplementary material for: Xanthohumol, a Prenylated Flavonoid from Hops, Induces Caspase-Dependent Degradation of Oncoprotein BCR-ABL in K562 Cells
Source: Antioxidants (Basel). 2019 Sep 16;8(9):402. doi: 10.3390/antiox8090402 (PMC6769755; doi:10.3390/antiox8090402)
Supplement: Supplementary file 1 [file antioxidants-08-00402-s001.pdf]

Xanthohumol, a Prenylated Flavonoid from Hops, Induces Caspase-Dependent Degradation of Oncoprotein BCR-ABL in K562 Cells

Xuxiu Lu [1], Jiajia Geng [1], Jinman Zhang [1], Jinlai Miao [2,3,*] and Ming Liu [1,2,4,*]

[1] Key Laboratory of Marine Drugs, Ministry of Education, School of Medicine and Pharmacy, Ocean University of China, Qingdao, 266003, China
[2] Laboratory for Marine Drugs and Bioproducts of Qingdao National Laboratory for Marine Science and Technology, Qingdao, 266237, China
[3] Key Laboratory of Marine Bioactive Substances, First Institute of Oceanography, MNR, Qingdao, 266061, China
[4] State Key Laboratory for Chemistry and Molecular Engineering of Medicinal Resources, Guangxi Normal University, Guilin, 541001, China
* Correspondence: lmouc@ouc.edu.cn (M.L.); miaojinlai@fio.org.cn (J.M.); Tel.: +86-532-8203-1980 (M.L.); +86-532-8896-7430 (J.M.)

Supplementary figures

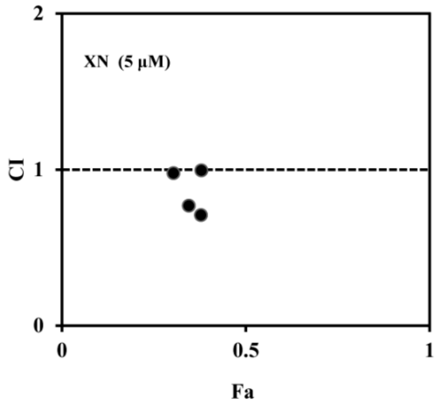

Figure 1. Evaluated the synergistic effect of XN combined with imatinib in K562/ADR cells. K562/ADR cells were treated with 5 μM of XN in the presence of 0.06 to 0.5 μM imatinib for 72 h. The CI was calculated by median effect plot analysis. Values for the combination index (CI) was calculated using software package Calcusyn (Biosoft, Cambridge, UK), which interpreted as follows: >1 antagonism, <1 synergism, and =1 additive. Fa represented the fractions of the affected cells (killed). All experiments were performed in three replicates (n = 3).

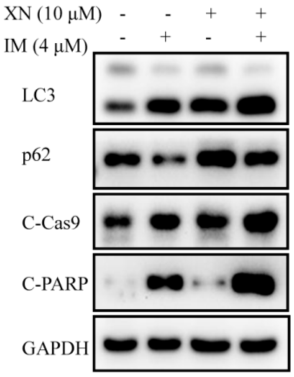

Figure 2. XN attenuates imatinib mediated autophagy and enhances apoptosis in K562/ADR cells. K562/ADR cells were treated with XN (10 μM) in the presence or absence of imatinib (4 μM) for 24 h. The expression of LC3, cleaved caspase9 (C-Cas9), and cleaved PARP (C-PARP) were determined by Western blotting.
